# To Investigate the Necessity of STRA6 Upregulation in T Cells during T Cell Immune Responses

**DOI:** 10.1371/journal.pone.0082808

**Published:** 2013-12-31

**Authors:** Rafik Terra, Xuehai Wang, Yan Hu, Tania Charpentier, Alain Lamarre, Ming Zhong, Hui Sun, Jianning Mao, Shijie Qi, Hongyu Luo, Jiangping Wu

**Affiliations:** 1 Laboratoire d'immunologie, Centre de recherche, Centre hospitalier de l'Université de Montréal (CRCHUM) – Hôpital Notre-Dame, Montréal, Québec, Canada; 2 Service de néphrologie, Centre de recherche, Centre hospitalier de l'Université de Montréal (CRCHUM) – Hôpital Notre-Dame, Montréal, Québec, Canada; 3 Institut national de la recherche scientifique (INRS) – Institut Armand-Frappier, Laval, Québec, Canada; 4 Department of Physiology, Jules Stein Eye Institute, David Geffen School of Medicine, University of California Los Angeles, Los Angeles, California, United States of America; MRC National Institute for Medical Research, United Kingdom

## Abstract

Our earlier study revealed that STRA6 (stimulated by retinoic acid gene 6) was up-regulated within 3 h of TCR stimulation. STRA6 is the high-affinity receptor for plasma retinol-binding protein (RBP) and mediates cellular vitamin A uptake. We generated STRA6 knockout (KO) mice to assess whether such up-regulation was critical for T-cell activation, differentiation and function. STRA6 KO mice under vitamin A sufficient conditions were fertile without apparent anomalies upon visual inspection. The size, cellularity and lymphocyte subpopulations of STRA6 KO thymus and spleen were comparable to those of their wild type (WT) controls. KO and WT T cells were similar in terms of TCR-stimulated proliferation *in vitro* and homeostatic expansion *in vivo*. Naive KO CD4 cells differentiated *in vitro* into Th1, Th2, Th17 as well as regulatory T cells in an analogous manner as their WT counterparts. *In vivo* experiments revealed that anti-viral immune responses to lymphocytic choriomeningitis virus in KO mice were comparable to those of WT controls. We also demonstrated that STRA6 KO and WT mice had similar glucose tolerance. Total vitamin A levels are dramatically lower in the eyes of KO mice as compared to those of WT mice, but the levels in other organs were not significantly affected after STRA6 deletion under vitamin A sufficient conditions, indicating that the eye is the mouse organ most sensitive to the loss of STRA6.

Our results demonstrate that 1) in vitamin A sufficiency, the deletion of STRA6 in T cells does no affect the T-cell immune responses so-far tested, including those depend on STAT5 signaling; 2) STRA6-independent vitamin A uptake compensated the lack of STRA6 in lymphoid organs under vitamin A sufficient conditions in mice; 3) STRA6 is critical for vitamin A uptake in the eyes even in vitamin A sufficiency.

## Introduction

During T-cell immune responses, naive T cells are activated by stimuli through TCR in the company of co-stimulation signals, and undergo multiple rounds of proliferation before entering the differentiation phase, after which they become effector T cells. The expression of many molecules is modulated during activation and differentiation stages, with some of them playing pivotal regulatory roles, while others exert support and house-keeping functions to cope with increased metabolic demands. We undertook unbiased exploration with DNA microarray analysis of molecules up- or down-regulated in T cells within the first 16 h after stimulation by anti-CD3 with a view to identifying those that are critical in the early T-cell activation stage. A group of molecules with the highest levels of altered expression in activated T cells was chosen, with resting T cells as reference, and verified by Northern blotting analysis. STRA6 (stimulated by retinoic acid gene 6) is among those that have been validated. We generated STRA6 gene knockout (KO) mice to assess the significance of its up-regulation in T-cell activation and, consequently, T-cell immune responses.

At the outset of our investigation in 2004, no function was ascribed to STRA6, a 74-kDa protein with multiple transmembrane domains that was first identified in retinoic acid-stimulated P19 embryonic carcinoma cells upon retinoic acid stimulation [1]. In 2007, Kawaguchi et al. used an unbiased technique to identify STRA6 as a specific cell-surface receptor for plasma retinol binding protein (RBP) and showed that STRA6 mediates cellular vitamin A uptake from holo-RBP (RBP/vitamin A complex) in bovine retinal pigment epithelium cells [2]. STRA6-mediated vitamin A uptake from holo-RBP is coupled to intracellular proteins as confirmed by several independent studies [1]–[5], and its mechanism in coupling to specific intracellular proteins has been elucidated [4]. Pasutto et al. [6] observed that mutations in STRA6 correlated with many eye, heart, diaphragm and lung malformations as well as mental retardation in Matthew-Wood syndrome in humans, corroborating its reported roles in vitamin A uptake by cells as vitamin A is vital in organogenesis. Recent reports indicate that single nucleotide polymorphisms or mutations in STRA6 gene are correlated with the congenital eye malformations microphthalmia, anophthalmia and coloboma [7], [8] as well as Matthew-Wood syndrome [9]. Genetic null mutation of STRA6 in mice results in significant retinoid reduction in the retinal pigment epithelium and neurosensory retina, diminished visual responses and eye morphology, although the last-mentioned defect is not as serious as in patients with STRA6 mutations [10].

There is a report suggesting that STRA6 is not only a vitamin A transporter but can also function as a cytokine receptor. Upon binding with holo-RBP, STRA6 is phosphorylated at tyrosine residue 643, which, in turn, recruits and triggers JAK2 and STAT5 activation [11].

The ascribed roles of STRA6 in vitamin A transport and the STAT5 signalling pathway are certainly relevant to T-cell activation and function. Retinoids are known to modulate Th1 (T helper 1), Th2, Th17 and reglulatory T (T_reg_) cell development and function [12]–[17]. At the molecular level, it has been demonstrated that retinoic acid opens up the FoxP3 promoter tertiary structure for activated FoxP3 transcription [18]. RARα can interact with STAT5a and b [19], which are critical molecules in the signaling pathway of a key T activation cytokine IL-2 [20].

Vitamin A is absorbed from dietary nutrients. There are several possible modes of vitamin A transport to cells in different organs. Vitamin A in the diet can be transported to liver cells and other cell types in the form of chylomicron-bound retinyl ester [21], [22]. The liver is the primary storage site for vitamin A in the form of all-trans-retinyl ester, which can be reverted to vitamin A [22]. As alluded to above, vitamin A associates with RBP in blood, and such complexes can deliver vitamin A to cells via the RBP receptor STRA6 [2]. Transthyretin can associate with vitamin A-bound RBP, and such coupling serve to prevent renal filtration of the holo-RBP [23], Recently, Alapatt et al. [24] discovered a second RBP receptor, a STRA6 homologue called RBPR2. RBPR2 is expressed in the liver, intestines, fatty tissues, and spleen. Like STRA6, RBPR2 is fully capable of binding to RBP and transporting vitamin A into cells. As vitamin A is hydrophobic, it should also be able to diffuse through cell membranes without any specific receptors.

The relative contribution of STRA6 to vitamin A cellular import in lymphoid organs has not been evaluated and is a secondary goal of our study.

In this study, we demonstrated that STRA6 KO mice were vital and fertile, manifesting no apparent anomalies in their lymphoid organs and T cell-dependent immune responses under vitamin A sufficient conditions. Intracellular vitamin A concentrations in lymphoid organs, such as the thymus and spleen of the KO mice were comparable to those of WT controls, although the vitamin A content in cells from KO eyes was significantly lower that that from the WT eyes. The implications of these data are discussed.

## Materials and Methods

### RT-qPCR

STRA6 mRNA in cells and tissues from KO, heterozygous and WT mice was measured by RT-qPCR. Total RNA was extracted with TRIzol® (Invitrogen, Carlsbad, CA, USA) and then reverse-transcribed with Superscript II™ reverse-transcriptase (Invitrogen). The forward and reverse primers were 5′-AGG CAT CTG AGA ATG GAA GCC AGA-3′ and 5′-AGC AGA ACC AGG AAC GAC AGT GAA-3′, respectively. A 184-bp product was detected with the following amplification program: 95°C×15 min, 1 cycle; 94°C×15 s, 55°C×30 s, 72°C×30 s, 35 cycles. β-actin mRNA levels were measured as internal controls; the forward and reverse primers were 5′-TGGTACCACAGGCATTGTGAT-3′ and 5′-TGATGTCACGCACGATTTCC CT-3′, respectively, with the same amplification program as for STRA6 mRNA. The data were expressed as ratios of STRA6 and β-actin signals.

### Generation of STRA6 KO mice

**Figure 1:**
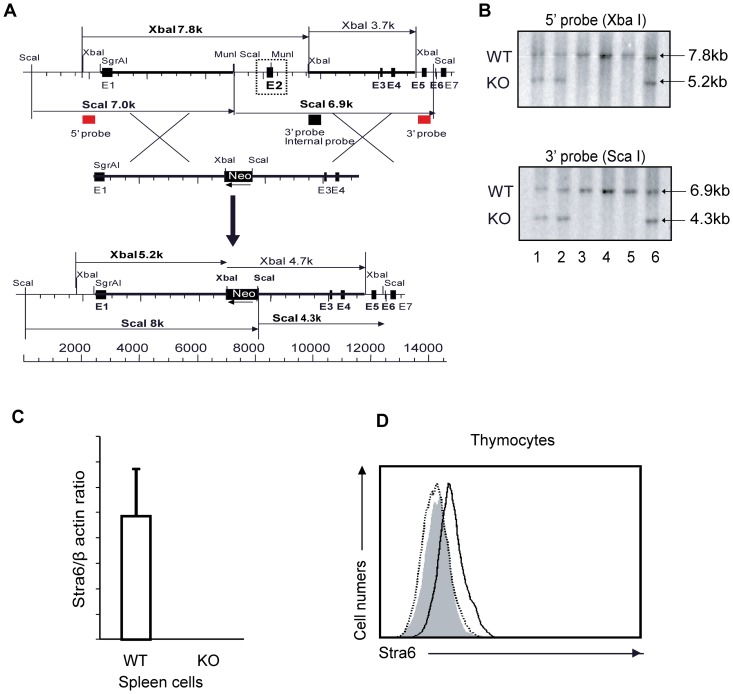
STRA6 mRNA expression in organs and activated T cells. STRA6 mRNA in organs (A) and activated total spleen T cells (B) was measured by RT-qPCR. WT spleen total T cells were cultured in wells coated with solid-phase anti-CD3 mAb and anti-CD28 mAb (0.5 µg/ml and 4 µg/ml, respectively, for coating) for the durations indicated. The cells were then harvested and their STRA6 mRNA levels measured by RT-qPCR. Samples were in triplicate for RT-qPCR, and means ± SD of ratios versus β-actin signals are reported. Experiments were conducted twice, and representative data are illustrated.

A PCR fragment amplified with the STRA6 cDNA sequence served as a probe to isolate genomic BAC DNA clone 7O8 from the RPCI-22 129/sv mouse BAC genomic library. The targeting vector was constructed by recombination [25] and routine cloning methods using an 11-kb STRA6 genomic fragment from clone 7O8 as the starting meterial. A 2.7-kb MunI-XbaI genomic fragment containing exon 2 was replaced by a 1.1-kb Neo cassette from pMC1Neo-Poly A flanked by 2 diagnostic restriction sites, XbaI and ScaI, as illustrated in Figure 1A. The final targeting fragment was excised from its cloning vector backbone by Not I digestion and electroporated into R1 embryonic stem (ES) cells for G418 selection [26]. The targeted ES cell clones were injected into C57BL/6 blastocysts. Chimeric male mice were mated with C57BL/6 females to establish mutated STRA6 allele germline transmission.

Southern blotting with probes corresponding to the 5′ and 3′ sequences outside the targeting region, as illustrated in Figure 1A (red squares), were used to screen for gene-targeted ES cells and eventually to confirm gene deletion in mouse tail DNA. With the 5′ probe, the targeted allele should present a 7.8-kb XbaI band, and the WT allele, a 5.2-kb XbaI band. With the 3′ probe, the targeted allele should present a 6.9-kb ScaI band, and the WT allele, a 4.3-kb ScaI band (Fig. 1A).

PCR was adopted for routine genotyping of the targeted allele(s). The following PCR conditions were applied: 4 min at 94°C, followed by 35 cycles of 30 s at 94°C, 30 s at 60°C, and 30 s at 72°C, with final incubation at 72°C for 10 min. The KO forward primer 5′- GCG TCA CCT TAA TAT GCG AAG TG-3′ and reverse primer 5′-CAA GAA GTC CGT GGC TGA GTC TA-3′ detected a 400-bp fragment from the targeted allele. The WT forward primer 5′-TCT CCC AGG TCT GGT TTG AG-3′ and reverse primer 5′-TTA GGG CAA CAC CCT ACT GG-3′ detected a 197-bp fragment from the WT allele.

The KO mice were backcrossed to the C57BL/6 background for 8 generations and then used for experimentation. All mice were housed under specific pathogen-free conditions and fed with mouse chow (Teklad Global 2018, Teklan Diets, Madison, WI) containing 15 IU/g Vitamin A. The mice had access to water and chow *ad libitum*. The mouse organs and tissues were retrieved after the mice were euthanatized by i.p. injection of 100 µl of Euthanyl (pentobarbital sodium, 120 mg/ml containing 1% lidocaine). The same method of euthanasia was used for unwanted heterozygous mice or extra unused mice. The studies were approved by the Institutional Animal Protection Committees of the CRCHUM and INRS-IAF.

### Flow cytometry

Single cell suspensions from the thymus, spleen and lymph nodes were prepared and stained immediately or after culture with antibodies (Abs) against CD4, CD8, CD25, CD19, B220, CD69 and STRA6. In some experiments, intracellular proteins, such as FoxP3, IFN-γ, IL-4, IL-17, and TNF-α, were detected after the cells were pre-stained with Abs against cell surface antigens fixed with BD Cytofix/Cytoperm™ solution (BD Biosciences, San Diego, CA) and then stained with monoclonal Abs (mAb) against intracellular antigens. The Abs deployed for flow cytometry are listed in Table 1. Flow cytometry analysis of the stained cells are described in our previous publications [27]–[30].

**Table 1:** Antibodies and reagents for flow cytometry.

*Antibody*	*Supplier*
PE-donkey anti-goat IgG	R & D Systems
APC-rat anti-mouse CD25 (clone PC61)	BD Biosciences
FITC-rat anti-mouse CD25 (clone 7D4)	BD Biosciences
PE-rat anti-mouse CD4 (clones GK1.5 and H129.19)	BD Biosciences
PerCP-rat anti-mouse CD4 (clone RM4-5)	BD Biosciences
biotin-rat anti mouse CD8b (clone 53-5.8)	BD Biosciences
APC-Cy7- anti-mouse B220 (clone RA3-6B2)	BD Biosciences
PE- or APC-hamster anti-mouse CD3ε(clone 145-2C11)	BD Biosciences
biotin- or FITC-rat anti-mouse CD44 (clone 1M7)	BD Biosciences
FITC- or PE-rat anti-mouse CD8α (clone 53-6.7)	BD Biosciences
APC-rat anti-mouse CD8α (clone H57-597)	BD Biosciences
PE-rat anti-mouse IL-17A	BD Biosciences
PE- and APC-rat anti-mouse IFN-γ	BD Biosciences
PE- and APC-rat anti-mouse IL4 mAbs	BD Biosciences
PerCP-streptavidin and 7-Amino-actinomycin D (7-AAD)	BD Biosciences
APC-Cy7-Streptavidin™	BioLegend
APC-rat anti-mouse TNF-α (clone MP6-XT22)	eBioscience (San Diego, CA)
FITC-rat anti-mouse IFN-γ (clone XMG1.2)	eBioscience
APC-rat anti-mouse IL17A (clone eBio17B7)	eBioscience
APC-rat anti mouse/rat Foxp3 (clone FJK-16s) mAbs	eBioscience
PE-Cy7-streptavidin	eBioscience
intracellular antigen fixation buffer	eBioscience
10× permeabilization buffer	eBioscience
APC-Cy™ PE-rat anti-mouse CD25 (clone PC61)	Cedarlane Laboratories Ltd (Burlington, Ontario, Canada)
Goat anti-mouse STRA6 Ab	Everest Biotech (Upper Heyford, Oxfordshire, UK)

Flow cytometry was also employed to assess lymphocytic choriomeningitis virus (LCMV)-specific T cells. Synthetic peptides gp_33–41_: KAVYNFATC (LCMV-GP, H-2D^b^); np_396–404_: FQPQNGQFI (LCMV-NP, H-2D^b^); gp_276–286_: SGVENPGGYCL (LCMV-GP, H-2D^b^); and gp_61–80_: GLNGPDIYKGVYQFKSVEFD (LCMV-GP, I-A^b^) were purchased from Sigma-Genosys (Oakville, Ontario, Canada). PE-gp_33–41_, PE-np_396–404_ and PE-gp_276–286_ H-2D^b^ tetrameric complexes were synthesized in-house and used at 1/100 dilution as previously described [26]. These MHC-tetramers were used to detect LCMV-specific CD8^+^ T cells on day 8 post LCMV infection. Briefly, splenocytes were first stained with PE-gp_33–41_, PE-np_396–404_ or PE-gp_276–286_ tetramers for 30 minutes at 37°C, followed by staining with FITC-rat anti-mouse CD8α and APC-rat anti-mouse CD62L mAbs at 4°C for another 20 minutes. 7-AAD was used for exclusion of dead cells. After washing, cells were fixed in 0.5% paraformaldehyde and samples were analyzed by flow cytometry. One million splenocytes from LCMV-infected mice were seeded in single wells of 96-well round-bottomed plates. They were maintained in 5% RPMI-1640 supplemented with 100 units/ml interleukin-2, 10 µg/ml brefeldin A, 10 µM gp_33–41_ or gp_61–80_ peptide. After 5 h of incubation at 37°C, the cells were stained with PE-conjugated rat anti-mouse CD8α or CD4 mAbs and 7-AAD. They were then fixed, permeabilized and stained with APC-labeled rat anti-mouse TNF-α and FITC-labeled rat anti-mouse IFN-γ mAbs. IFN-γ and TNF-α-secreting T cells were counted by flow cytometry [31].

### T-cell proliferation in vitro and in vivo after being transferred to sub-lethally-irradiated mice

Spleen cells were loaded with carboxyfluorescein succinimidyl ester (CFSE; 5 µM for 5 mins), and then cultured in the presence of soluble hamster against mouse CD3 mAb (clone 2C11; 0.5 µg/ml) [27], [28], [32], [33]. After 3 days, CFSE fluorescence of the CD4 and CD8 subpopulations was analyzed by flow cytometry for TCR-stimulated proliferation. T-cell homeostatic expansion was evaluated by i.v. injection of 5×10^6^ CFSE-loaded spleen cells into C57BL/6 recipients 5 h after sub-lethal irradiation (650 Rad). On day 5, the CFSE fluorescence of CD4 and CD8 cells from the spleen and LN was studied by flow cytometry.

### *In vitro* Th1, Th2, Th17 and T_reg_ cell polarization

*In vitro* Th and T_reg_ cell differentiation was conducted as follows [27], [34]. Naïve CD4 T cells (CD4^+^CD62L^+^CD44^low^) were isolated from KO or WT mouse spleens with MagCellect Mouse Naïve CD4^+^ T cell Isolation kits (R & D Systems). T cell-depleted WT spleen cells were irradiated at 3000 Rad and used as feeder cells. The naïve CD4 cells (0.1×10^6^/well) were mixed with the feeder cells (0.5×10^6^/well) and cultured in 96-well plates in the presence of soluble anti-CD3ε mAb (clone 145-2C11, 2 µg/ml; BD Biosciences). Cultures were supplemented with recombinant mouse IL-12 (10 ng/ml; R & D Systems) and anti-IL-4 mAb (10 µg/ml; R & D Systems) for the Th1 condition; recombinant mouse IL-4 (20 ng/ml; R & D Systems), and anti-IL-12 mAb (10 µg/ml; BD Biosciences) and anti-IFN-γ mAb (10 µg/ml; R & D Systems) for the Th2 condition; recombinant mouse IL-6 (20 ng/ml; R & D Systems), recombinant human TGF-β1 (5 ng/ml; R & D Systems) and anti-IL-4 and anti-IFN-γ mAbs (10 µg/ml for each; R & D Systems) for the Th17 condition; recombinant human TGF-β1 (5 ng/ml; R & D Systems), and anti-IL-4 and anti-IFN-γ mAb (10 µg/ml; R & D Systems) for the T_reg_ condition.

### LCMV infection

LCMV clone 13 was obtained from Dr. R.M. Zinkernagel (University of Zurich, Zurich, Switzerland). Viral stock was propagated *in vitro*, and viral titers were quantified by focus-forming assay [31]. Mice were infected by the i.v. route with 2×10^6^ focus-forming units of LCMV clone 13. They were sacrificed 8 days post-infection, and their spleens were harvested for primary immune response analysis.

### Glucose tolerance tests

The KO and WT mice were fasted for 16 h and injected i.v. with D-glucose (2 mg/g body weight) in PBS. Blood samples from the tail vein were taken at 5, 15, 30, 60, and 90 min after the injection for glucose measurements with a glucose meter (Bayer, Toronto, Ontario).

### Measurement of serum and intracellular vitamin A and retinyl ester concentrations by high-pressure liquid chromatography (HPLC)

Serum and tissue samples, collected in a dark, cold room, were stored at −80°C until their analysis. Retinoids were extracted by homogenizing tissues in a butanol-acetonitrile mixture (1∶1) with a tissue/solvent ratio of 200 mg/700 µl, in Eppendorf tubes on ice by 5 30-s pulses with 1-min intervals. K_2_HPO_4_ solution (6.89M) was added to the tubes in proportion to the homogenized mixture (20 µl for 900 µl homogenized mixture). For retinoid extraction from sera, 200-µl butanol-acetonitrile mixture (1∶1) was added to 200-µl serum, and the mixture was vortexed for 1 min; 20 µl K_2_HPO_4_ solution (6.89M) was then added to the mixture before 30-s vortexing. The tissue and serum samples thus prepared were centrifuged for 20 min at 14,000 *g* at 4°C. Cleared supernatants were passed through Spin-X filters (0.45 µm pore size; Costar, Batavia, Illinois, USA) at 14,000 *g* for 10 min at 4°C. For retinyl ester measurement, the samples prepared as aforementioned before the step of filtration were vacuum-dried and re-dissolved in 100% methanol, followed by centrifugation at 14,000 g for 10 min. The supernatants were then analyzed by HPLC.

Vitamin A in extracts was quantified by HPLC in an ÄKTA Purifier (Model UPC10; GE Healthcare, Baie d'Urfé, Quebec, Canada) and reverse-phase column (μ-RPC C2/C18 ST 4.6/100; GE Healthcare). Samples (100 µl) were eluted with a linear gradient from 100% eluent A (acetontrile∶water = 65∶35) to 100% eluent B (acetontrile∶water = 90∶10) in 5-column volumes at a flow rate of 1 ml/min. Both eluates contained 10 mM ammonium acetate. Vitamin A was detected at 313 nm wave-length. Its characteristic retention volume was identified with pure Vitamin A from Sigma (Oakville, ON, Canada) as a standard. Areas under the curves were computed by UNICORN5.11 software (GE Healthcare). The sensitivity of the assay was 250 ng.

Retinyl ester in extracts was quantified by HPLC in an Eclipse XDB-C18 reverse-phase column (4.6×150 mm, 5 µm, Agilent, Santa Clara, CA). Samples (200 µl) were eluted with a linear gradient from 100% methanol to 100% ethyl acetate in 5 column volumes at a flow rate of 1 ml/min. Retinyl ester was detected at 324 nm wavelength. Its characteristic retention volume was identified with retinyl palmitate (Sigma) as standard. Areas under the curves were computed by Agilent LC software. Sensitivity of the assay was 1.5 ng.

### Statistics

Student's t tests were employed to analyze statistical differences between WT and KO mice for their lymphoid organ weight and cellularity, and for their retinoid contents (retinol and retinyl esters) in different organs. ANOVA was used to compare the glucose tolerance between WT and KO mice.

## Results

### STRA6 expression in different organs and activated T cells

**Figure 2:**
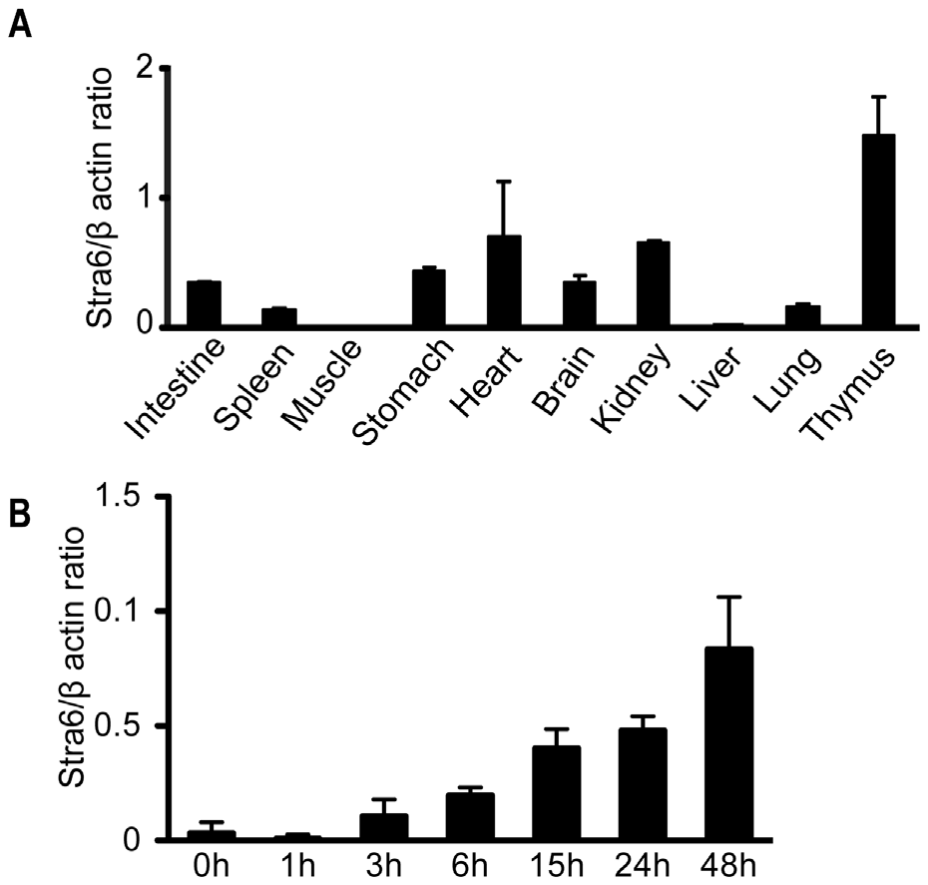
Generation of STRA6 KO mice. *A. Targeting strategy for generating STRA6 KO mice.* The red squares on the 5′ and 3′ sides of the mouse STRA6 WT genomic sequence represent the sequences serving as probes for genotyping by Southern blotting. *B. Genotyping of STRA6 mutant mice.* Tail DNA was digested with XbaI, and analyzed by Southern blotting (top panel), with the 5′ probe whose location is indicated in A. A 7.8-kb band representing the WT allele and a 5.2-kb band representing the recombinant allele are indicated by arrows. Similarly, tail DNA was digested with ScaI, and analyzed with the 3′ probe (bottom panel). A 6.9-kb band representing the WT allele and a 4.3-kb band representing the recombinant allele are indicated by arrows. *C. Absence of STRA6 mRNA expression in STRA6 KO splenocytes.* STRA6 mRNA levels from WT and KO splenocytes were analyzed by RT-qPCR. The RT-qPCR samples were in triplicate and the results are expressed as ratios of STRA6 versus β-actin mRNA signals with means ± SD indicated. The experiments were conducted twice and representative data of one experiment are shown. *D. Absence of STRA6 protein expression in KO thymocytes.* WT and KO thymocytes were stained with goat anti-mouse STRA6 Ab, and analyzed by flow cytometry. The shaded area is the isotypic Ab staining control using WT thymocytes. The thick line represents WT thymocytes stained with anti-STRA6 Ab, and the dotted line, KO thymocytes stained with anti-STRA6 Ab. The experiments were carried out three times and a representative histogram is shown.

STRA6 mRNA expression was assessed by RT-qPCR. Among the organs and tissues examined, the thymus had the highest expression level, followed by the heart and kidneys (Fig. 2A). STRA6 expression in the spleen was moderate. The skeleton muscles and liver had barely detectable STRA6 mRNA. The STRA6 mRNA expression levels in the lung, liver, spleen and kidney assessed by our RT/qPCR was consistent with Northern results reported previously by Bouillet [1]. High STRA6 expression in the thymus suggested that it might have some critical functions in T-cell development and T-cell function. As depicted in Figure 2B, STRA6 expression in resting spleen T cells (0 h) was modest, consistent with values of the whole spleen. The expression was augmented with in 3 h after T-cell activation by TCR cross-linking, and reached a peak at 48 h. This result corroborates our initial DNA microarray data, through which STRA6 was found upregulated during T-cell activation.

### Generation of STRA6 KO mice

To evaluate the roles of STRA6 in the immune system in general and T cell-mediated immune responses in particular, we produced STRA6 KO mice. The targeting strategy is illustrated in Figure 1A. Germline transmission was confirmed by Southern blotting of tail DNA (Fig. 1B). With the 5′ end probe, the WT allele after XbaI digestion presented a 7.8-kb band, and the KO allele, a 5.2-kb band (Fig. 1B, upper panel). With the 3′ end probe, the WT allele after ScaI digestion presented a 6.9-kb band, and the KO allele, a 4.3-kb band (Fig. 1B, lower panel). WT (mice 3, 4, and 5) and heterozygous mice (mice 1, 2 and 6) were thus identified. Mouse 1 was backcrossed to the C57BL/6 background for 8 generations, and then used in the experiments described hereafter.

To ascertain whether STRA6 gene deletion results in its lack of expression, we measured STRA6 mRNA in spleen cells by RT-qPCR. STRA6 mRNA was detectable in WT but not in KO spleen cells (Fig. 1C). The lack of STRA6 expression in KO cells at the protein level was confirmed by flow cytometry, as STRA6 was detectable in WT but not KO thymocyte surface (Fig. 1D).

### Normal lymphoid organs and lymphocyte subpopulations in STRA6 KO mice

**Figure 3:**
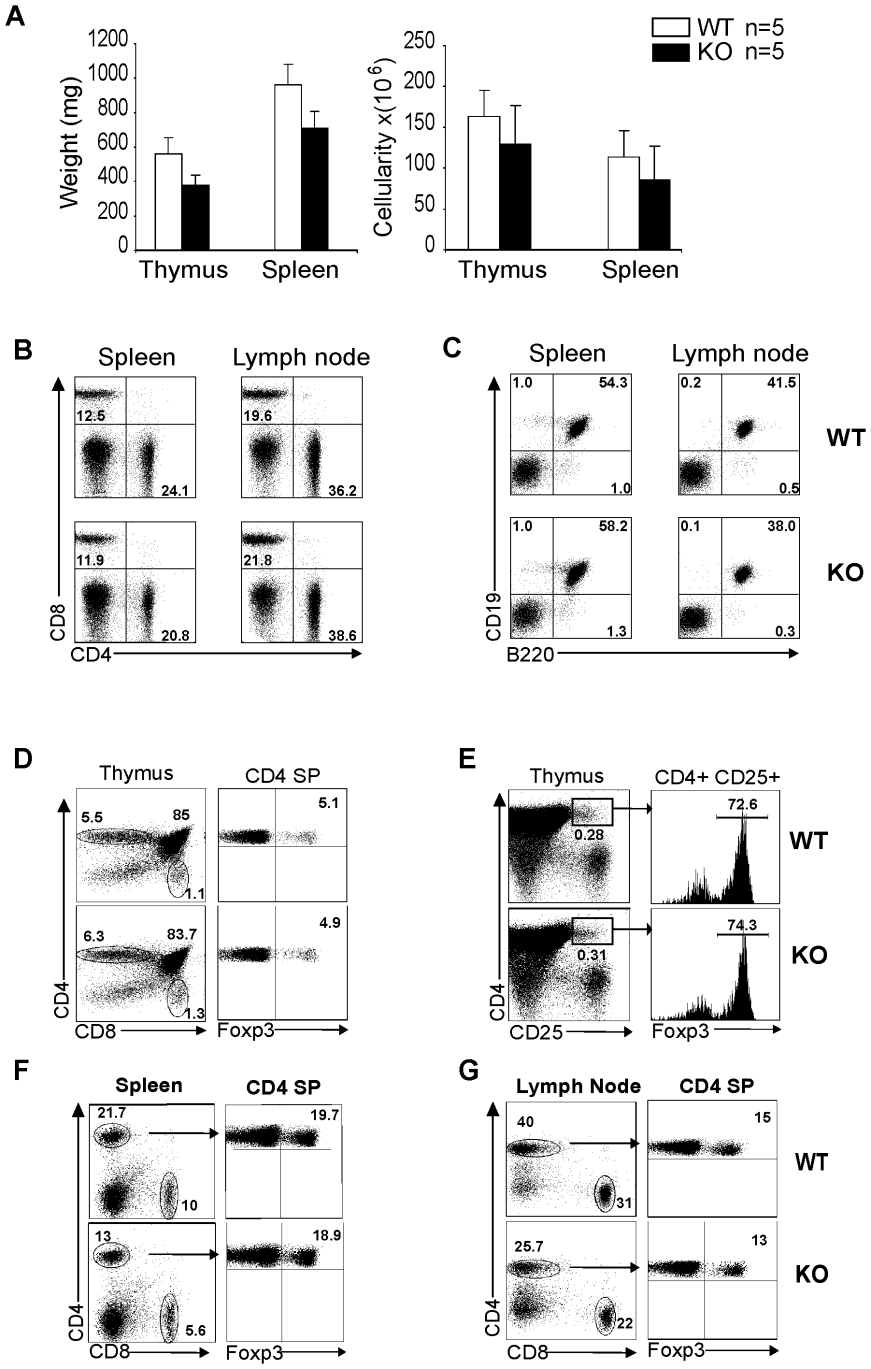
STRA6 KO mice presented normal lymphoid organs and lymphocyte subpopulations. *A. Weight and cellularity of WT and KO thymi and spleens.* Mouse number (n) in each group is shown. No significant difference is detected in weight and cellularity between KO and WT organs (*p*>0.01, paired Student's *t* test). *B. CD4 and CD8 T-cell subpopulations in WT and KO spleens and LN.* Splenocytes and LN cells were analyzed by 2-color flow cytometry for percentages of CD4 and CD8 T cells. *C. B cell populations in the spleen and LN of WT and KO mice.* Splenocytes and LN cells were analyzed by 2-color flow cytometry for percentages of CD19^+^/B220^+^ B cells. *D and E. Normal thymocyte subpopulations and endogenous T_reg_ cells in KO thyme.* CD4/CD8 double-negative, CD4/CD8 double-positive, CD4 single-positive and CD8 single-positive cells and CD4^+^CD8^−^FoxP3^+^ T_reg_ cells in KO and WT thymi were analyzed by 3-color flow cytometry (D). CD4^+^CD25^+^FoxP3^+^ T_reg_ cells in the WT and KO thymi were also analyzed by 3-color flow cytometry (E). *F and G. CD4^+^CD8^−^FoxP3^+^ T_reg_ cells in KO and WT spleens and LN*. CD4^+^CD8^−^FoxP3^+^ T_reg_ cells in KO and WT spleens (F) and LN (G) were analyzed by 3-color flow cytometry. The experiments in B through G were conducted more than 3 times, and representative histograms are presented. Percentages of relevant populations are indicated.

STRA6 KO mice were viable and fertile with no apparent anomalies upon visual inspection. Weight and cellularity of the KO thymus and spleen were comparable to those of WT mice (Fig. 3A). T-cell (CD4^+^ plus CD8^+^ versus non T-cell (CD4^−^CD8^−^) subpopulations, and CD4 versus CD8 T-cell subpopulations in the spleen and LN of WT and KO mice showed no consistent differences (Fig. 3B). The percentages of B cells (CD19^+^B220^+^) in the spleen and lymph nodes in WT and KO mice were also similar (Fig. 3C). In KO thymi, the percentages of CD4 single-positive and CD8 single-positive, CD4CD8 double-positive cells and CD4^+^/FoxP3^+^ T_reg_ cells were comparable to those in WT thymi (Fig. 3D). The comparable percentages of T_reg_ cells in the thymi of WT and KO mice were confirmed by the measurement of FoxP3^+^ cells among CD4^+^CD25^+^ thymocytes (Fig. 3E).

In the periphery, the percentages of FoxP3^+^ T_reg_ cells among CD4 cells in the spleen (Fig. 3F) and lymph nodes (Fig. 3G) from WT and KO mice were also similar.

These results show that STRA6 KO mice have normal lymphoid organ and T-cell development.

### Normal activation, proliferation and differentiation of STRA6 KO T cells

**Figure 4:**
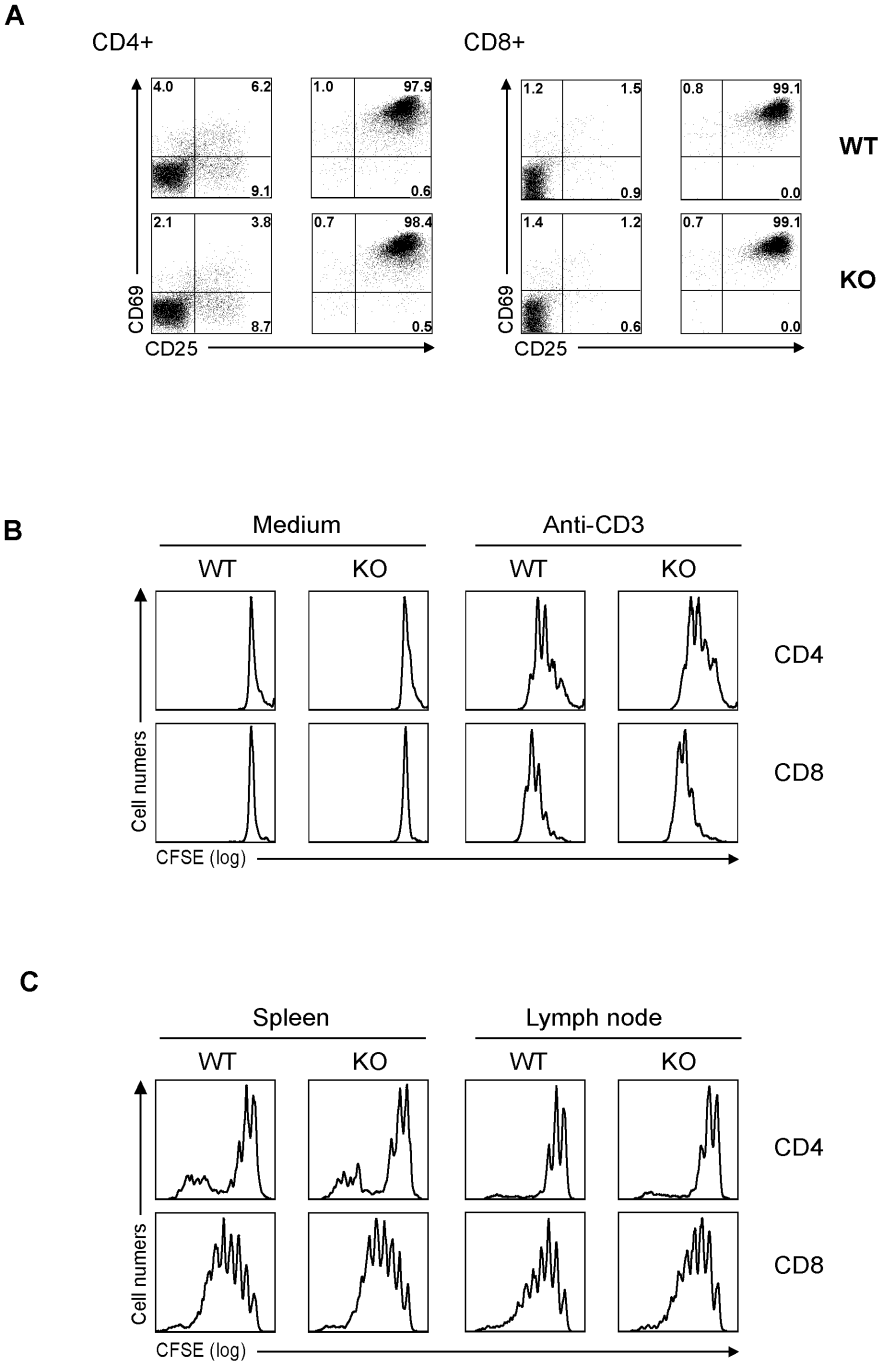
Normal activation and proliferation of KO T cells. *A. Normal activation marker CD69 and CD25 expression on KO T cells.* Total spleen cells were stimulated overnight by soluble anti-CD3 mAb (0.5 µg/ml). CD69 and CD25 expression on CD4 (left panel) and CD8 (right panel) T cells was measured by 3-color flow cytometry. *B. Normal proliferation of KO CD4 and CD8 cells upon TCR activation in vitro.* Total spleen cells were loaded with CFSE and then stimulated with soluble anti-CD3 mAb (0.5 µg/ml). The cells were harvested after 72 h, and stained for CD4 and CD8; CFSE levels in these cells were analyzed by 3-color flow cytometry. *C. KO CD4 and CD8 cells present normal homeostatic expansion in vivo.* Five million CFSE-loaded spleen cells were injected i.v. into sub-lethally irradiated (650 Rad) C57BL/6 recipients 5 h after the irradiation. On days 6, the CFSE fluorescence of CD4 and CD8 cells from the spleen and LN was analyzed by flow cytometry. All experiments in this figure were conducted twice or more, and representative histograms are shown.

KO and WT T cells were stimulated by solid-phase anti-CD3 mAb for 16 h. The activation markers CD25 and CD69 in CD4 and CD8 T cells were quantified by flow cytometry. KO and WT T cells showed similar up-regulation of these markers (Fig. 4A). To assess T-cell proliferation, KO and WT T cells in total spleen cells were loaded with CFSE and stimulated by soluble anti-CD3 mAb. After 3 days, their proliferation was assessed by flow cytometry. CD4 and CD8 KO T cells proliferated like their WT counterparts, as shown in Figure 4B. To measure T-cell homeostatic expansion, spleen T cells were loaded with CFSE and then injected into sub-lethally-irradiated syngeneic recipients. The proliferation of these transferred KO CD4 and CD8 cells in recipient spleens and LN during the 5 days after the injection was measured based on their CFSE content according to flow cytometry. As shown in Figure 4C, the cells from WT and KO mice proliferated similarly in vivo. Therefore, KO T-cell proliferation, whether caused by TCR stimulation in vitro or homeostatic expansion in vivo, was not defective.

**Figure 5:**
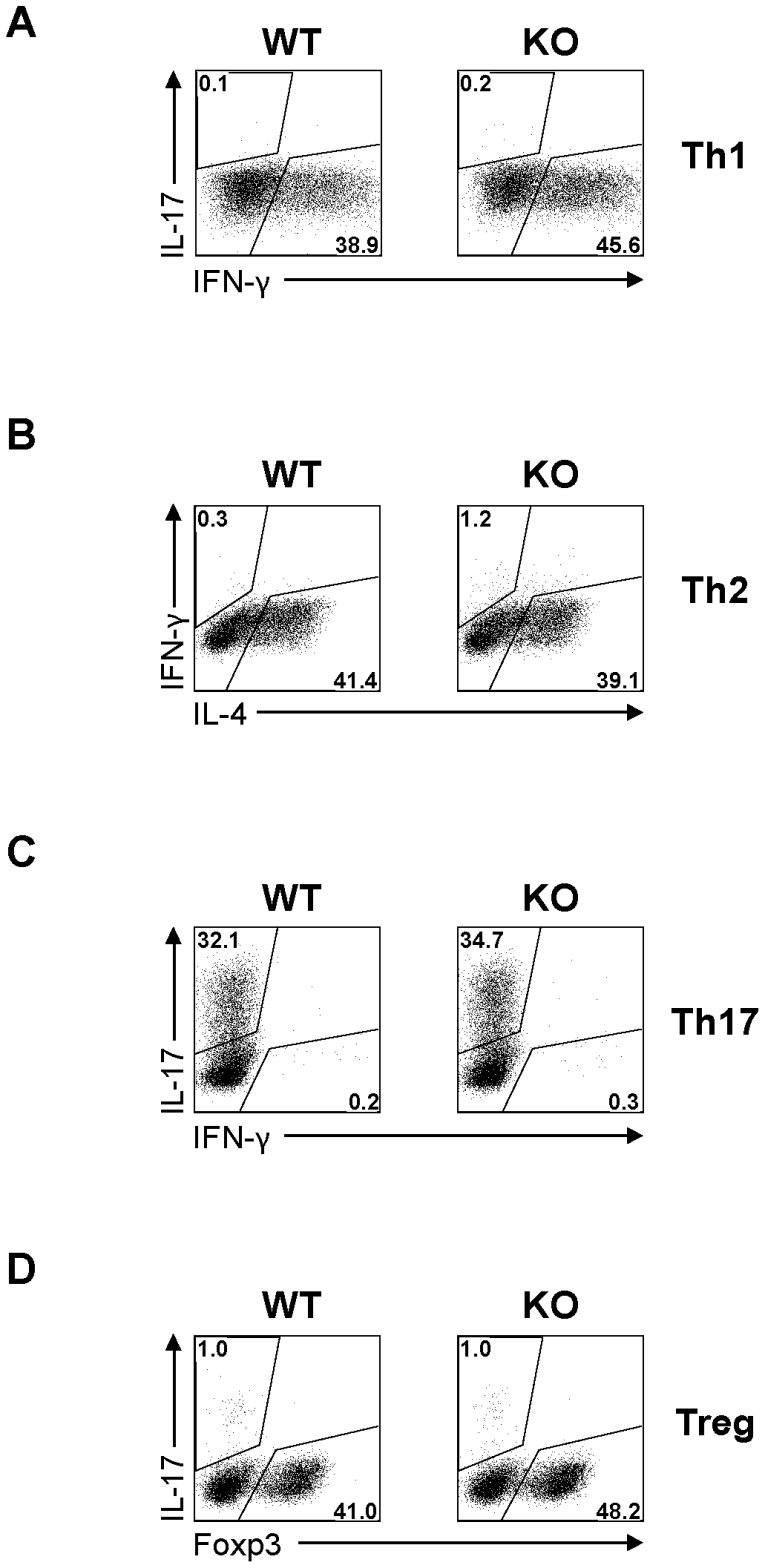
Normal differentiation of STRA6 KO CD4 cells in vitro. Naïve CD4 cells were cultured under conditions favouring Th1 (A), Th2 (B), Th17 (C) and T_reg_ (D) cell differentiation. Their intracellular cytokine or FoxP3 expression was quantified by flow cytometry on day 3 for Th1, Th17 and T_reg_ cells, and on day 5 for Th2 cells. Experiments were repeated more than 3 times, and representative histograms are shown.

When KO and WT naïve CD4 cells were cultured under Th1, Th2, Th17 and T_reg_ conditions, they achieved comparable Th1, Th2, Th17 and T_reg_ cell percentages (Fig. 5), indicating normal differentiation of naïve KO CD4 cells into these subpopulations.

### The effect of STRA6 deletion in anti-LCMV immune responses in vivo

**Figure 6:**
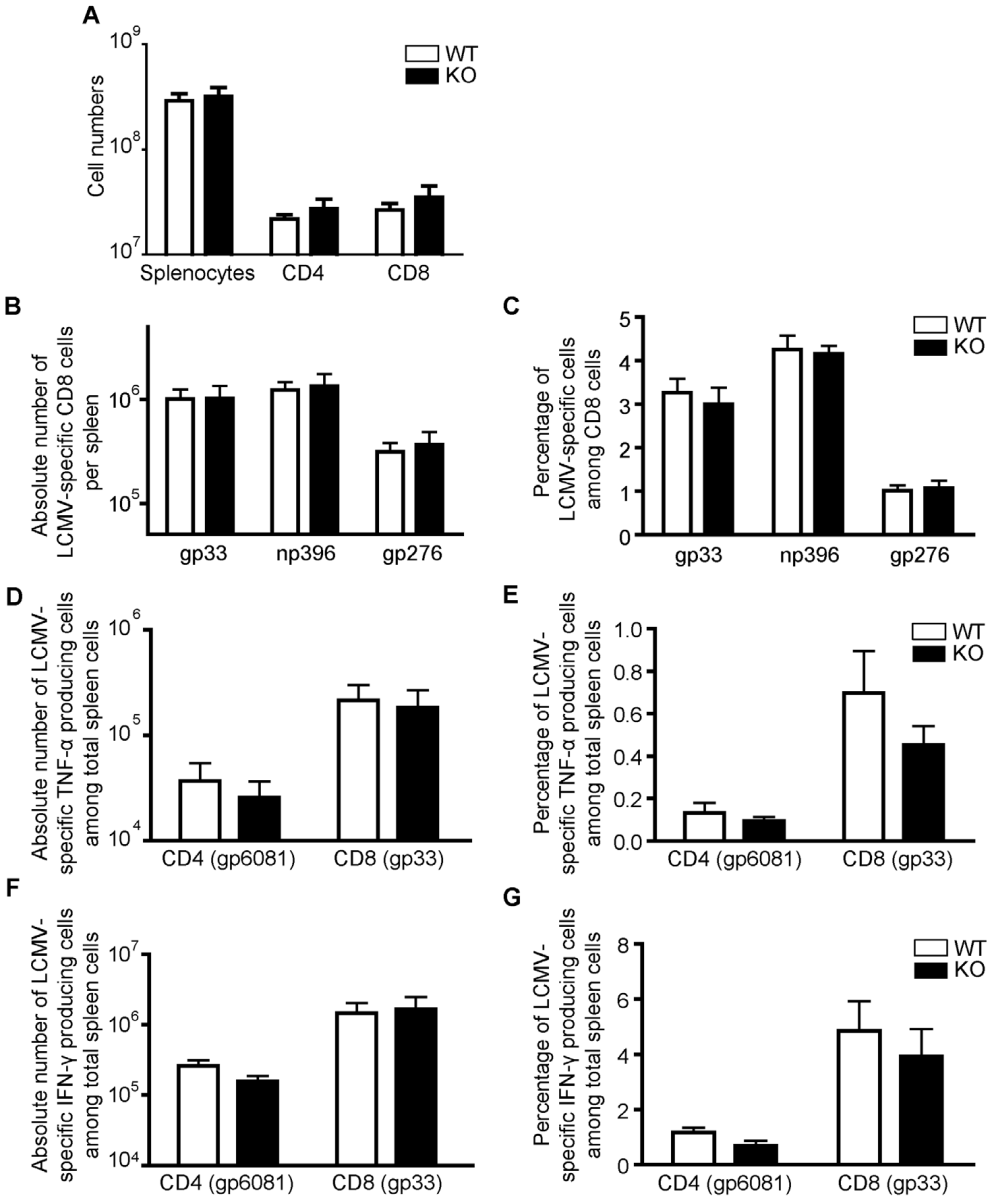
Normal in vivo anti-LCMV immune responses of STRA6 KO mice. *A. Spleen cell numbers on day 8 after LCMV infection.* Means ± SD of absolute numbers of total splenocytes, CD4^+^ cells, and CD8^+^ cells in spleens of WT littermate control (n = 4) and KO (n = 4) mice on day 8 post-LCMV infection are presented. *B and C. LCMV-specific CD8 cells on day 8 post-LCMV infection.* On day 8 post-infection, the absolute numbers of gp33, np396 and gp276 tetramer-positive CD8 T cells per spleen (B) and the percentage of gp33, np396 and gp276 tetramer-positive cells among CD8 cells (C) were measured by flow cytometry. Means ± SD of data from 4 pairs of WT littermate control and STRA6 KO mice are presented. *D and E. LCMV-specific TNF-α-producing CD4 and CD8 cells on day 8 post-LCMV infection.* The absolute number of TNF-α-producing LCMV-specific CD4 cells (gp61-specific) and CD8 cells (gp33-specific) per spleen (D) and percentage (E) of these cells among total spleen cells of KO and WT mice on day 8 post-LCMV infection. Means ± SD of data from 4 pairs of STRA6 KO mice and WT littermate controls are shown. *F and G. Virus-specific IFN-γ-producing CD4 and CD8 cells on day 8 post-LCMV infection.* The absolute number of TNF-α-producing LCMV-specific CD4 cells (gp61-specific) and CD8 cells (gp33-specific) per spleen (D) and percentage (E) of these cells among total spleen cells of KO and WT mice on day 8 post-LCMV infection. Means ± SD of data from 4 pairs of STRA6 KO mice and WT littermate controls are shown. The results in this figure are analyzed by Student's t test. No significant difference was found between WT and groups.

The function of STRA6 KO T cells *in vivo* was evaluated in the LCMV infection model. As illustrated in Figure 6A, the number of total splenocytes, and CD4 and CD8 cells on day 8 post-infection (8 dpi) presented no significant differences in WT and KO mice (Fig. 6A). The absolute numbers (Fig. 6B) and relative percentages (Fig. 6C) of LCMV-specific tetramer-positive (gp33^+^, np396^+^ and gp276^+^) CD8 cells in virus-infected mice were all increased in comparison to uninfected control C57BL/6 mice (data not shown), but there were no significant differences between KO and WT mice with regard to these parameters. The absolute numbers and relative percentages of LCMV-specific TNF-α-producing CD4 (gp61) and CD8 cells (gp33) (Figs. 6D and 6E), and LCMV-specific IFN-γ-producing CD4 and CD8 cells (Figs. 6F and 6G) in KO mice were comparable to those in WT controls. These results indicate that the STRA6 deletion had no discernable effect on anti-LCMV immune responses.

### Normal glucose tolerance in STRA6 KO mice

**Figure 7:**
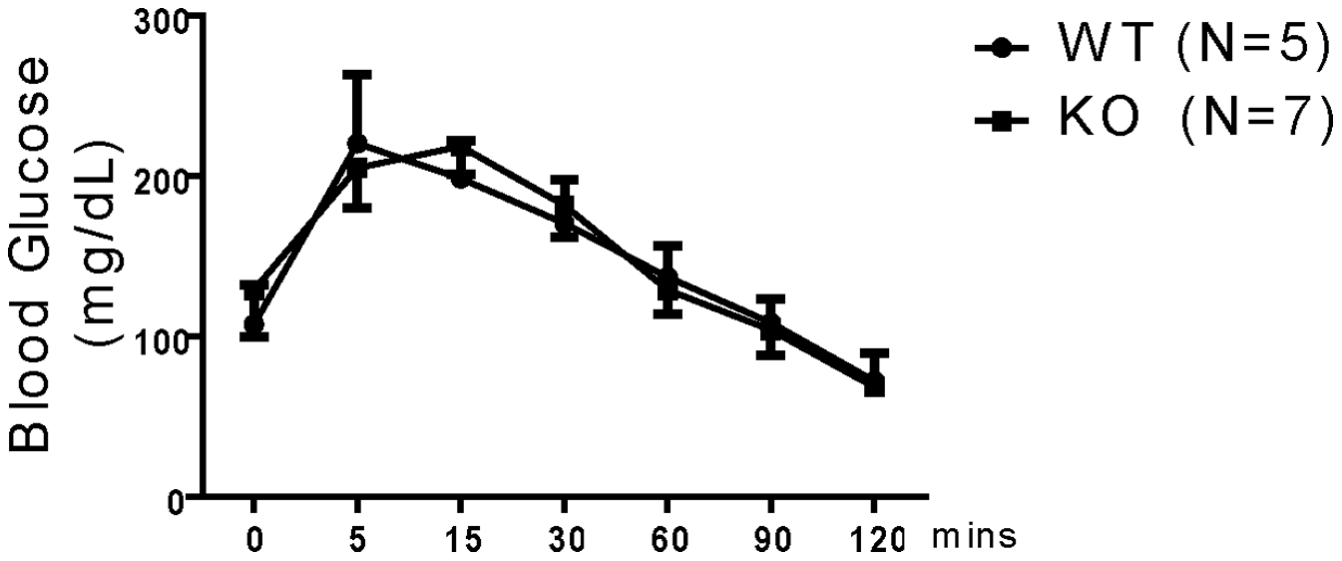
Glucose tolerance of KO and WT mice. WT (n = 5) and KO (n = 7) mice were fasted for 16 h, and then injected i.p. with D-glucose (2 mg/g body weight). Blood glucose was measured at different time points from the time of injection until 120 min. Means ± SD of glucose levels (mg/dL) are reported. No statistical significant difference is observed between the KO and WT groups (ANOVA).

One report suggests that STRA6 stimulation by RBP induces the expression of SOCS3, which inhibits insulin signaling [12]. We assessed the glucose tolerance of KO mice, and found that, KO and WT mice showed no significant difference in glucose tolerance (Fig. 7), suggesting that in the absence of STRA6, the insulin signaling of the KO mice on a normal diet is not enhanced.

### Organ retinyl ester and retinol levels in STRA6 KO mice

As vitamin A has been reported to play an important role in immune regulation [12]–[17], a lack of immunological phenotype so-far tested in the KO mice prompted us to examine vitamin A contents of lymphoid organs as well as several other organs including the eyes. Vitamin A is stored in organs predominantly in the form of retinyl ester, which is a lipid and can reach high concentrations. A minor stored form is retinol bound to CRBP, and the retinol content in the cells is limited by the availability of CRBP. Retinyl esters and retinol and can be quickly converted to each other inside the cells. We thus measured the contents of both retinyl esters and retinol in these organs.

**Figure 8:**
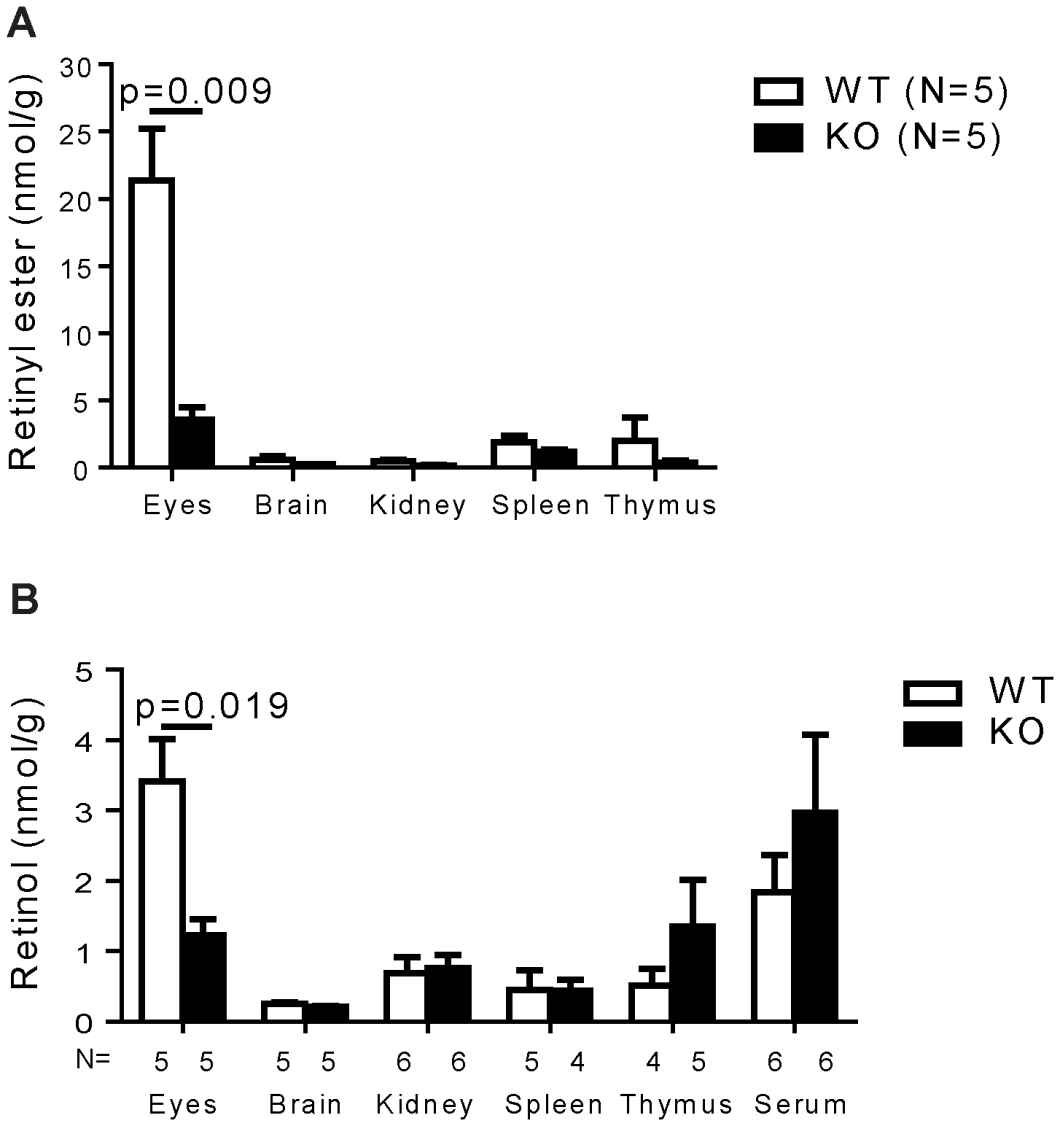
Intracellular retinoid contents in lymphoid and other organs of STRA6 KO mice were comparable to those of WT mice. Retinoid (retinyl ester, A; retinol, B) contents (nmol/gram tissue or nmol/ml serum) of the eyes, brain, kidney, spleen, thymus, spleen, thymus and sera from KO and WT mice were measured by HPLC. The mouse numbers (n) per group are indicated. The results are expressed as means+SD. The *p*-values are indicated when significant (Student's *t* test).

As shown in Figure 8A, the WT and KO spleen and thymus had no significant difference in their retinyl ester contents, nor did the brains and kidneys. The retinyl ester contents in WT eyes were much higher than that of other organs, and the contents in the KO eyes were significantly lower compared to those of the WT counterparts. WT or KO blood had no detectable retinyl ester (data not shown). Retinyl ester is known to be high in the blood only right after a meal enriched in vitamin A.

The retinol levels in these WT and KO organs were of the same pattern as retinyl ester, although at much lower levels (Fig. 8B; note the scale difference). The KO spleen, thymus, kidney and brain had no significant difference in retinol contents compared to their WT counterparts. Unlike retinyl ester, the retinol was detectable in the sera, but was of similar levels in WT and KO sera. The eyes contained the highest levels of retinol. KO eyes presented significantly lower levels of retinol than the WT counterparts.

These data indicate that under a vitamin A sufficient condition, lymphoid organs still take up vitamin A without STRA6 in mice. This explains the lack of immunological phenotype in the KO mice. However, even under such a vitamin A sufficient condition, the eyes still heavily depend on SRAT6 for vitamin A uptake, as they are the organ with the highest vitamin A demand.

## Discussion

STRA6 is a receptor of holo-RBP (i.e., vitamin A-bound RBP) for cellular vitamin A uptake. STRA6 is up-regulated after T-cell activation. In this study, we generated STRA6 KO mice to assess whether such up-regulation was essential for T cell-mediated immune responses and STRA6's role in vitamin A uptake. Under a vitamin A sufficient condition, STRA6 KO mice developed normally and were fertile. Their T cells presented no signs of abnormality in terms of development, activation marker up-regulation, proliferation, and Th and T_reg_ cell differentiation. KO mice also had normal anti-LCMV immune responses. There was no significant difference in intracellular vitamin A content, in the forms of both retinyl ester and retinol, in lymphoid organs from WT and KO mice. However, even under the vitamin A sufficient condition, the KO eyes contained significantly lower amounts of retinyl ester and retinol, indicating a critical role of vitamin A uptake in this organ.

A caveat of whole organ retinoid analysis is that contribution of retinoid in the blood can affect the total retinoid levels. This is especially true if the organ is rich in blood, which contains RBP-bound vitamin A. Despite this caveat, whole organ retinoid analysis can be used as an approximation of cellular retinoid uptake. This is especially true as several of the organs we tested (i.e., the thymus, brain and eyes) are not blood rich. Moreover, sera had no detectable retinyl ester (data not shown); so the retinyl ester levels of the organs tested will not be upward influenced by the blood retinyl ester levels. The reduced vitamin A contents in the eyes of STRA6 KO mice is not unexpected, as the eyes have the highest concentrations of vitamin A among all the organs (Fig. 8) due to its heavy reliance on vitamin A for vision, and probably need all the capacities of vitamin A transport including the pathway of RBP/STRA6 to achieve this high vitamin A content, even in vitamin A sufficiency. Consistent to our findings, RBP KO mice have normal vitamin A levels in most of their organs, but a reduced one in the eyes [35].

Vitamin A and its metabolites – retinoic acids – are clearly required in immune responses [13]–[18], [36]. It is reported that in hepatocytes, holo-RBP triggers STRA6, leading to the activation of JAK2/STAT5 signaling pathway, which is also essential in the activation and function of immune cells. STRA6 is up-regulated within 24 h of T-cell activation (Fig. 1B). Is such up-regulation, or more fundamentally, the existence of STRA6, essential for T cell-mediated immune responses? We demonstrated that in STRA6 KO mice, a lack of STRA6 did not affect T-cell activation/proliferation/differentiation *in vitro* and anti-viral immune responses *in vivo* under a vitamin A sufficient condition. These observations suggest following possible and not necessarily mutually exclusive explanations: 1) STRA6 up-regulation/existence only becomes important for T-cell functions during vitamin A deficiency, when all capacities of vitamin A import to immune cells are required; 2) STRA6 homologue RBPR2 can compensate for STRA6 function in the immune cells, as this homologue is expressed in the spleen; also, retinyl esters bound to lipoproteins secreted by the small intestine can deliver vitamin A to peripheral organs under vitamin A sufficient conditions; 3) STRA6 plays a minimal role in modulating the JAK2/STAT5 signalling pathway in immune cells, and its upregulation after T cell activation has nothing to do with JAK2/STAT5 signaling; 4) We cannot exclude the possibility that STRA6 deletion might still affect certain T cell-mediated immune responses to some extent, but they have not been assessed in our experiments, or their magnitude was too small to be discerned by current assays; 5) Such up-regulation might be a parallel and irrelevant event during T-cell activation.

There is little systemic documentation on vitamin A sufficiency status in wild mammals in today's world. However, it is well-documented that vitamin A deficiency is prevalent in African and Southeast Asian populations, particularly affecting children and pregnant women, according to the World Health Organization [37], and such deficiency predisposes them to infectious diseases [38]. It is conceivable that, during evolution, mammals might have experienced vitamin A deficiencies in certain periods or regions in the world. Better cellular vitamin A transport will confer an evolutionary advantage to these animals with regard to but not restricted by their ability to cope with infectious diseases. If STAR6 is universally critical in all cell types for vitamin A uptake, its function should be revealed in immune responses in vitamin A deficiency. Experiments addressing this possibility are in progress.

STRA6 point mutations are found in some patients, with microphthalmia, anophthalmia, coloboma [7] and Matthew-Wood syndrome [referring to combinations of microphthalmia/anophthalmia, cardiac malformations, pulmonary dysgenesis, and diaphragmatic hernia; ref. 9]. In a study of 2 unrelated consanguineous families with malformation syndromes sharing anophthalmia and distinct eyebrows as common signs, homozygous mutations were identified in STRA6 [6]. Our STRA6 KO mice and those generated by Ruiz *et al.* [10] did not have dramatic phenotypes, such as a total absence of eyes, as seen in humans with STRA6 mutations.

Why cannot STRA6 KO in mice reproduce human disease phenotypes caused by STRA6 mutations? A simple explanation is that this is due to species differences. It is not unprecedented that gene mutations in mice and humans have very different phenotypes. For example, partial or complete loss of ABCA4 functions cause many blinding diseases in humans including retinitis pigmentosa, cone-rod dystrophy and Stargdardt macular dystrophy, but ABCA4 KO in mice does not cause blindness unless combined with a deletion of other genes such as RDH8 [39]–[41]. On the other hand, disease loci of microphthalmia and anophthalmia have been mapped to multiple chromosomes [42]–[45]. Patients with Matthew-Wood syndrome or malformation syndromes have quite large phenotype variations in terms of organ affliction and disease severity. Such observations suggest that these syndromes are not monogenic, and STRA6 mutation alone is not sufficient to evoke all such phenotypes. It could explain why no serious ophthalmic [13] or other organ malformations are apparent in STRA6 null mutation mice. If STRA6's major function is cellular vitamin A uptake, and human organ malformation syndromes are mainly caused by a lack of available intracellular vitamin A, it would support the notion that STRA6 only plays a minor role in cellular vitamin A uptake in vitamin A sufficiency, especially in organs other than the eyes. Unless other routes of cellular vitamin A uptake such as those mediated by RBPR2 or by retinyl esters bound to lipoproteins are simultaneously compromised, vitamin A in the cells of most, if not all, organs vitamin A contents will remain in the normal range, and the organs will develop and function normally in vitamin A sufficiency. However, significant phenotype might be revealed in vitamin A deficiency. This hypothesis is supported by results from RBP KO mice. These KO mice are fertile and have no organ abnormality other than the vision phenotype [46]–[48], as is the case of STRA6 KO mice when fed with a vitamin A sufficient diet. However, they manifest severe systemic phenotype of embryonic lethality under a vitamin A deficient condition [49], [50]. Consistently, in mouse embryo culture where is no retinyl ester pathway, RBP knockdown also causes severe developmental defects [51].

In summary, we conclude that, under normal dietary conditions, mouse lymphoid organ development, T-cell activation and differentiation, including T_reg_ cell development, and anti-LCMV responses, could proceed normally in the absence of STRA6 under vitamin A sufficient conditions.
